# The Relationship between Survival Sex and Borderline Personality Disorder Symptoms in a High Risk Female Population

**DOI:** 10.3390/ijerph14091031

**Published:** 2017-09-08

**Authors:** Jerreed Ivanich, Melissa Welch-Lazoritz, Kirk Dombrowski

**Affiliations:** Department of Sociology, University of Nebraska-Lincoln, Lincoln, NE 68588, USA; melissa.lynn.welch@gmail.com (M.W.-L.); kdombrowski2@unl.edu (K.D.)

**Keywords:** homeless, borderline personality disorder, survival sex, high risk populations

## Abstract

Engaging in survival sex and mental illness are overrepresented within homeless populations. This article assesses the relationship between symptoms of borderline personality disorder (BPD) and engaging in survival sex among homeless women. One hundred and fifty-eight homeless women completed surveys on self-reported BPD symptomology and sexual history. Bivariate and multivariate analyses conducted in this study provided insights into the association of experiencing BPD symptoms and engaging in survival sex. Results indicate that some symptoms of BPD are robustly correlated with engaging in survival sex among homeless adult women. Implications for service agencies and others working with at-risk female populations are discussed.

## 1. Introduction

Engaging in survival sex (the exchange of sex for money, goods, housing, or other material items needed to remain personally and socially viable) has been linked to many negative outcomes, including victimization, suicide attempts, and sexually transmitted diseases [1]. Although prevalence estimates vary based on sampling frame, location, resources, time of year, and other factors, some studies have found that homeless and runaway populations have the highest rates of survival sex participation of all at-risk groups [2]. Across multiple studies, individuals who are homeless have regularly shown higher odds of engaging in survival sex compared to those who are not homeless [1], with reported rates of 11% to 41% [3,4,5], compared to rates of 3.5%–5.0% in the general population [6].

Complicating this picture is the fact that individuals experiencing an episode of homelessness are at higher risk for a host of other negative outcomes. Homeless individuals see increased odds of coming into contact with the criminal justice system [7,8,9], experiencing mental illness [10,11,12], being a victim of sexual exploitation [10,13,14], and acquiring sexually transmitted diseases [15]. Potential contributing causes are many. Research has emphasized the lack of safety that comes from not having a stable place to live, which in turn exposes individuals to increased risk of negative experiences [16,17,18].

Other findings draw specific attention to the fact that homeless experience is shaped along gender lines [19,20,21]. Homeless females are often driven to homelessness and runaway decisions for very different reasons than their male counterparts [22]. The experience on the street is also lived differently for women compared to men. For instance, the odds of being a victim living on the streets is amplified for females compared to the odds prior to living on the street [19,23]. Additionally, homeless females are also burdened with having more problems with some mental health concerns (i.e., internalizing disorders) and substance use [20,24]. Although these studies suggest reasons why being female may be associated with elevated risks for survival sex participation during periods of homelessness, few studies have directly examined the causes of survival sex participation among adult homeless women. This is notable given the relatively prominent role that survival sex plays in research on homeless children and young adults [25,26]. This paper seeks to remedy this gap by exploring the role of mental illness—with a focus on symptoms associated with borderline personality disorder—in contributing to participation in survival sex exchanges among homeless adult women.

Among the more prominent mental health concerns for homeless adult women is the increased rate of borderline personality disorder (BPD) [27]. Data suggests that homeless populations see rates of BPD of approximately 20% [28]. These rates are more than tenfold higher than the 1.3% of non-homeless individuals experiencing BPD [29,30]. Further, research has established a relationship between dysfunctional sexual behaviors and BPD. The majority of these studies have focused on complex emotional needs that influence of sexual relationships (i.e., attachment, intimacy, and trust) that frequently appear as manifestations of BPD symptoms [31,32]. Further, women who meet criteria for BPD (when compared to those who do not) are significantly more likely to have stressful and strained sexual relationships over time [32]. Even short of full BPD diagnosis, individuals who manifest the symptoms of BPD have been shown to have an extreme intolerance for abandonment, being alone, and often engage in dysfunctional sexual behaviors to maintain attachment [33]. Methods of maintaining attachments and avoiding being alone may be linked to BPD via manifestations of impulsivity (i.e., acting out irrationally to maintain connection) and abandonment. In all of these cases, common BPD symptoms provide potential links between the presence (or risk) of mental illness with sexual practices. The question we seek to answer here is whether, in the case of homeless women, such factors may influence their recourse to exchange sex—driven by the financial and personal insecurity that accompany homelessness.

Understanding national or regional prevalence rates of BPD among homeless populations is problematic for two major reasons. First, BPD is highly associated with treatment seeking, and fewer resources have been dedicated to understanding BPD outside of treatment settings when compared to other personality disorders [34]. Second, conducting research among homeless populations is inherently difficult due to low visibility, high mobility, and frequent reluctance to participate in research. Scholars have noted differences between research and clinical diagnostic routines [35] for BPD that can also complicate field diagnoses. Further, disentangling the relationships between impulsivity, abandonment, other previous diagnoses, and substance abuse in a non-clinical setting is difficult [21]. Given these issues, we follow the lead of researchers that have focused on BPD symptoms when diagnosis is not available or applicable [36]. This strategy has shown that women that engage in more sexually risky behaviors display higher levels of impulsivity and abandonment [31,33]—common symptoms and diagnostic criteria for BPD [37]. Similar approaches among men who have sex with men have shown that risky sexual behavior accompanies common symptoms of BPD [38]. Our approach follows the work of Northey et al. [39] in viewing sexual implications of BPD symptomology among a non-clinical population, contributing to an emerging focus on the relational aspects of this mental illness [40].

## 2. Current Study

The Diagnostic and Statistical Manual of Mental Disorders, 5th Edition (DSM-5) [41] indicates that nine different dimensions be considered in clinical review of borderline personality disorder. The nine dimensions are: frantic efforts to avoid real or imagined abandonment, anger, sudden mood shifts, emptiness, identity disturbance, dissociative behavior, suicide, impulsivity, and unstable relationships. Although scholars have noted the association between BPD and sexual impulsivity, scholars have yet to explore the nine different dimensions of BPD for independent associations with survival sex for homeless adult female populations. The goal of the current study is to examine the relationship between BPD symptoms and survival sex among a sample of homeless women. For the purposes of this paper, those who engage in sex for survival tactics (i.e., food, shelter, and protection) and in trading sex for money will be included in the definition of survival sex—similar to that of [1]. These data were collected among homeless women with the specific aim of collecting information on BPD. The results of this article provide unique insights into the association between the different dimensions of BPD and engaging in survival sex. Thus, the results of this study are a valuable contribution for mental health service providers and service providers.

**Hypothesis** **1.** Engaging in survival sex will be associated with symptoms of BPD.

**Hypothesis** **2.** Abandonment and impulsivity will remain significantly associated with engaging in survival sex after controls are introduced.

## 3. Data and Methods

Data were collected from women in three cities (i.e., Omaha, Nebraska (NE), Pittsburgh, Pennsylvania (PA), and Portland, Oregon (OR)) as part of a pilot study designed to test a sampling design and measures for use with homeless women. The women in shelters were by definition considered to be homeless. Women interviewed at meal locations or outdoor locations self-identified as homeless and were screened regarding meeting the federal McKinney Act definition. The interviews were conducted between August 2010 and May 2011 to account for the effects of seasonal changes on sampling outcomes. Prior to the interview, the women were screened for mental status and sobriety before they were asked to give informed consent.

A total of 561 potential participants were selected to participate from summer 2010 through the winter of 2011. Two hundred and seven women (36.9%) were missed, either because they did not respond to the letters at shelters or because project staffers were unable to make contact in meal or outdoor locations. Exactly 147 contacts (26%) were to ineligible individuals (due to not identifying as homeless (N = 64), gender (N = 33), age (N = 38), or being selected at a meal or outdoor location when they had utilized a shelter within the past week (N = 11)). Forty-four women were contacted and eligible but refused (21.5% of the contacted eligible women). Exactly 163 women were contacted, eligible, and interviewed for this project (78.8% of the contacted, eligible women). Five of these women were interviewed during both the first (summer) and second (winter) round of sampling selection, and thus were administered the identical survey twice. Their second interviews were removed from the dataset, leaving a total of 158 women who completed at least one section of the interview. Therefore, the sample used here consisted of 141 women from shelters, 16 women from meal locations, and 1 woman from an outdoor location. For each completed interview, the respondent received $20 for their participation. All participants were recruited, interviewed, and agreed to participation with all processes and considerations approved by the Principle Investigator’s home university institutional review board, in accordance with the Declaration of Helsinki (IRB Approval #20100110352FB).

### 3.1. Measures

#### 3.1.1. Focal Independent Variables

The DSM-5 indicates that nine different dimensions be considered in clinical review of borderline personality disorder. The nine dimensions are abandonment, anger, sudden mood shifts, emptiness, identity disturbance, dissociative behavior, suicide, impulsivity, and unstable relationships. The diagnostic interview for BPD was a structured diagnostic interview completed in paper and pencil. The diagnostic standardized interview was based on each of the dimensions of the DSM-IV BPD symptoms. For a detailed discussion of the standardized BPD interview, see Whitbeck et al. [42]. Each interview contained multiple questions on the independent dimension of BPD in the form of a root question (e.g., “During the past two years, have you felt angry a lot of the time?”). Each of the root questions would be followed up with questions regarding the frequency in which the root question happened in the past two years. All of the root questions for each dimension were combined and summed. From the newly created summed variable, a dichotomous measure was created for each of the nine dimensions. For the dichotomous measures, 0–3 counts were set to 0 (low) and 4+ were set to 1 (high). This produced nine dichotomous (low count and high count) variables for each dimension of BPD.

#### 3.1.2. Dependent Variable

Survival sex is a dichotomous variable of homeless women that have engaged in survival sex. This measure was created using four different questions; each asked the respondent if they had ever had sex in exchange for some form of survival need. The “needs” considered for criteria for the survival sex measure included exchanging sex for money, food, shelter, and protection. The combination of these four survival needs yielded 35 women (22%) that had used sex for survival.

#### 3.1.3. Demographic Variables

Race, age, education and years homeless were all included as control variables as they have proven to be important constructs to include in the homeless literature [2,5]. Race is a dichotomous variable (white/non-white). Age is a continuous variable with a minimum age of 19 and a maximum age of 54 years (mean = 38.89). Education was measured by splitting the years of school attainment into two groups—12 years of education or less (0) and 13 years of education or more (1). Time homeless is a continuous variable summing the number of years a participant had experienced homeless throughout her lifetime, ranging from 1–31 years.

### 3.2. Analytic Approach

All analyses were conducted using Stata. This study first provides descriptive information of the demographic and clinical characteristics of the data. Second, we present bivariate results showing the association of each independent BPD symptom and engaging in survival sex. Multivariate logistic regression analyses were used to calculate the odds ratios for the BPD symptoms that are associated with engaging in survival sex, net the effect of age, time spent homeless, education, and race.

## 4. Results

### 4.1. Demographic and Clinical Characteristics

Table 1 presents the descriptive statistics for all of the variables included in the study, including the dependent variable (survival sex), the independent variables, and the control variables. As shown in Table 1, 23.8% of women in the sample indicated that they have engaged in survival sex at some point in their life. The nine dimensions of BPD are included in Table 1, and 39% of women in the study fell in the high count category for mood shifts and nearly 29% of women fell into the high count for anger. All other dimensions of BPD range from 13.43% to 39.26% of the sample falling into the high count category. The sampled women had spent an average of 4.76 years experiencing homelessness, more than half (58.67%) had more than a high school education, and were 43.9% white.

### 4.2. Bivariate Results

Table 2 presents the cross tabulations between the independent variables and control variables, and the dependent variable. Six of the nine included variables showed significant associations with survival sex at the bivariate level. Specifically, there was very strong support for abandonment—15.12% of individuals who reported low levels of abandonment engaged in survival sex, whereas 41.46% of individuals who reported high levels of abandonment had engaged in survival sex. Similar significant patterns emerge when looking at the suicidal symptoms, where 19.42% of the low count category had engaged in survival sex and 41.67% of the high count category engaged in survival sex. Anger, identity, impulsivity, and unstable relationships all showed significant difference between those who fell into the high count category and the low count category in terms of engaging in survival sex. The dimensions of BPD that did not have significant associations were mood shifts, emptiness, and dissociative behavior. No significant association was noted for education and race (white vs. non-white).

### 4.3. Multivariate Results

Table 3 presents the logistic regression models predicting the engagement in survival sex in odds ratios. Model 1 presents the logistic regression of each of the nine dimensions of BPD as dichotomous variables as predictors of survival sex. Results of Model 1 indicate that mood shifts were negatively associated with survival sex and impulsivity was positively associated with survival sex, when controlling for all other BPD dimensions. More specifically, having a high count of mood shifts was associated with a 0.679 lower odds of engaging in survival sex compared to having low counts of mood shifts. Having high counts of impulsivity was associated with 3.204 increased odds of engaging in survival sex relative to those who have low count in impulsivity, all else being constant.

Model 2 tests whether the results in Model 1 would remain significant when controlling for race, age, education, and years spent being homeless. It is noted that mood shifts was no longer significantly associated with survival sex when controlling for race, age, education, and years spent being homeless, yet impulsivity remained significant. In fact, the effect size increased from 3.2 to 4.2 once the control variables were included in the model. The only control variable to be significantly associated with engagement in survival sex was age. As individuals got older they were more likely to engage in survival sex.

**Figure 1:**
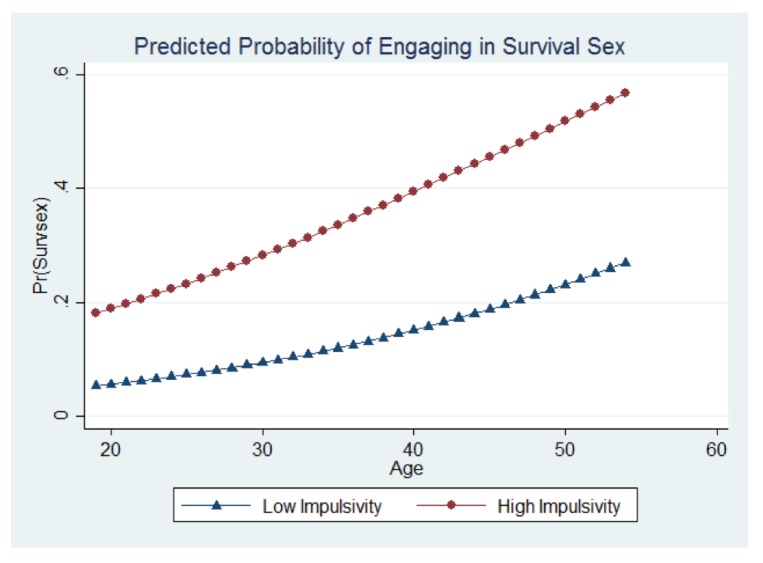
Predicted probability of engaging in survival sex.

Figure 1 presents the predicted probabilities of engaging in survival sex as a function of age and impulsivity, as these are the only significant predictors after controlling for other symptoms of BPD, education, race, and years homeless. Figure 1 shows that the older a woman is, the probability of engaging in survival sex became higher. Furthermore, high impulsivity increased the probability of engaging in survival sex. The average participant in our study was approximately 38 years old. Comparing the experiences of those at the average age across the two predicted probability groups (low impulsivity and high impulsivity) showed that at 38 years old, the predicted probability for a female that had a low count of impulsivity was approximately 0.15. For women of the same age but with a high level of impulsivity, the predicted probability increased to just under 0.40. The predicted probability of engaging in survival sex for a woman of the average age (38) with high counts of impulsivity compared to low counts of impulsivity was more than double.

## 5. Discussion

This is the first study to examine individual symptom classes of borderline personality disorder in relation to survival sex in a high-risk homeless population. We hypothesized that the nine individual symptom dimensions would be significantly associated with engaging in survival sex. We also hypothesized that impulsivity and abandonment would remain associated with survival sex after controlling for demographic factors. We found partial support for the first hypothesis. At the bivariate level, five of the nine symptoms were significantly associated with survival sex. Additionally, two of the nine symptoms were associated with survival sex in Model 1 of our logistic regression models. We also found partial support for our second hypothesis. While abandonment and impulsivity were both significantly associated with survival sex at the bivariate level, only impulsivity remained significant in the multivariate logistic regression models.

Overall, our findings are somewhat surprising. Past research has linked both impulsivity and abandonment to sexually risky behaviors [31,33]. As such, we expected to find both impulsivity and abandonment to be significantly associated with engaging in survival sex, yet we only found significant evidence linking impulsivity with engaging in survival sex. The clinical and diagnostic criteria for BPD inherently situates individuals that meet criteria for BPD as being attachment-seeking individuals [41]. Individuals in underprivileged states (i.e., homeless women) that have high count levels of impulsivity and abandonment were expected to be more likely to follow the theorized attachment-seeking and engage at higher odds in survival sex, yet this was not fully realized. These results suggest that impulsivity—over abandonment—warrants more detailed review in terms of theory and empirical support.

Given the results presented here, several implications for service providers are recommended. Due to the high association of impulsivity and age, service providers that work with homeless populations—especially homeless adult women—should be afforded training to understand and recognize the most at-risk group for intervention and assistance. For instance, older women that display high levels of impulsivity should be a high priority for intervening to prevent increased risk of engaging in survival sex. While work is being done to understand the services for trauma in homeless populations [43], more work is needed to explore intervention, local and onsite prevention, and training programs impacts for service providers addressing mental health needs for the people they serve.

### Limitations and Future Research

The primary limitation of this study is sample size. Although it is a small study, the data are from multiple cities and across multiple sites within the cities, which are strengths. This study is limited in that it did not test for other forms of risky sexual behavior outside of survival sex. We utilized cross-sectional data, and as such, a causal relationship between BPD symptoms and survival sex engagement was not asserted or tested. Of the 561 potential participants, we were unable to contact 207 (36.9%), and we cannot be certain what biases these missed contacts introduce into our findings. An additional limitation of our sampling design is that we limited the age range to age 54 years, so older women were not included.

In light of the limitations presented and the results of this study, continued efforts are needed to untangle the relationship between survival sex and borderline personality disorder. Longitudinal data should be utilized to understand the temporal relationship between survival sex and BPD symptoms. The temporal ordering of events is one critical component that is not present in this study, but may shed a great deal of light on the understanding of the interplay between these concepts. Lastly, social scientists that are engaged in the unique blend of behavioral and mental health research should expand resources and efforts in developing and disseminating reliable measures of BPD for non-clinical settings.

## 6. Conclusions

Despite the limitations, the present study utilizes a unique high-risk sample of women experiencing homelessness that is uniquely situated for exploring the association between both survival sex and BPD, which is not available in samples of the general public. Women who find themselves in periods of homelessness often do not have the resources or support to avoid engaging in survival sex. Furthermore, these women often are limited in their ability to seek help for medical conditions, including mental health. This translates to a disadvantaged population that needs additional support through housing initiatives and mental health care services. The current findings suggest that older individuals with high levels of impulsivity symptoms may be especially at risk for engaging in survival sex. Engaging in survival sex could have harmful consequences to a woman’s physical and mental health, and it may put her at risk for additional trauma.

## Figures and Tables

**Table 1:** Demographic and clinical characteristics (n = 158).

Variable	Percent	Std. Deviation
Survival Sex	23.81	0.427
Borderline Personality Symptoms ${}^{a}$		
Anger	28.89	0.455
Mood Shifts	39.26	0.49
Emptiness	23.88	0.428
Identity	14.18	0.35
Dissociative	15.67	0.365
Suicide	13.43	0.342
Abandonment	20.15	0.403
Impulsivity	29.1	0.456
Unstable Relationships	24.63	0.432
White	43.92	0.498
Age (19–54)	38.89	10.18
Education (% with 13+ years)	58.67	0.494
Years Homeless	4.76	4.802

${}^{a}$ Report of those who had high counts.

**Table 2:** Crosstabs between survival sex and categorical independent variables (n = 158).

Variable		% Who Engaged in Survival Sex	*p*-Value
Anger	Low	16.48%	0.003 ***
	High	41.67%	
Mood Shifts	Low	19.74%	0.208
	High	29.41%	
Emptiness	Low	22.68%	0.653
	High	26.67%	
Identity	Low	20.00%	0.015 **
	High	41.06%	
Dissociative	Low	22.22%	0.376
	High	31.58%	
Suicide	Low	20.00%	0.015 **
	High	47.06%	
Abandonment	Low	19.61%	0.031 **
	High	40.00%	
Impulsivity	Low	14.77%	0.000 ***
	High	43.59%	
Unstable Relationships	Low	21.05%	0.24
	High	31.25%	
Race	Non-White	26.83%	0.273
	White	19.05%	
Education	<12 years	23.73%	0.985
	13+	23.86%	

** *p* < 0.05; *** *p* < 0.01.

**Table 3:** Logistic regression models of borderline personality disorder (BPD) symptoms on survival Sex.

	Model 1	Model 2
Survival Sex		
Borderline Personality Symptoms ${}^{†}$		
Anger	3.241	2.824
Mood Shifts	0.679 **	0.686
Emptiness	0.322	0.246
Identity	4.845	4.959
Dissociative	0.51	0.544
Suicide	2.113	2.748
Abandonment	1.671	1.289
Impulsivity	3.204 **	4.193 **
Unstable Relationships	0.501	0.518
White		0.612
Age		1.059 *
Education		1.291
Years Homeless		1.035
*AIC*	137.4	137.3
*BIC*	165.8	182.4
*N*	158	158

* *p* < 0.1; ** *p* < 0.05; ${}^{†}$ Reference for each BPD symptoms is “low count”.

## Abbreviations

The following abbreviations are used in this manuscript:

BPD	Borderline Personality Disorder
